# Unveiling of Evolution Pattern for HY12 Enterovirus Quasispecies and Pathogenicity Alteration

**DOI:** 10.3390/v13112174

**Published:** 2021-10-28

**Authors:** Xiaoran Chang, Lisai Zhu, Junying Hu, Qun Zhang, Fuhui Zhang, Qian Lin, Xiaochun Gai, Xinping Wang

**Affiliations:** 1 College of Veterinary Medicine, Jilin University, Changchun 130062, China; changxr20@mails.jlu.edu.cn (X.C.); zhuls@haid.com.cn (L.Z.); jyhu19@mails.jlu.edu.cn (J.H.); zhangqun21@mails.jlu.edu.cn (Q.Z.); zhangfh21@mails.jlu.edu.cn (F.Z.); carolynxiaoxiao@163.com (Q.L.); gxc412318@163.com (X.G.); 2 Key Laboratory for Zoonoses Research, Ministry of Education, Changchun 130062, China

**Keywords:** HY12 enterovirus, EV-E, quasispecies, evolution pattern, mutation, selection

## Abstract

Enterovirus, like the majority of RNA viruses, evolves to survive the changeable environments by a variety of strategies. Here, we showed that HY12 virus evolved to alter its characteristics and pathogenicity by employing a non-synonymous mutation. Analyses of 5′UTR, VP1 and VP2 gene sequences revealed the existence of HY12 virus in an array of mutants defined as quasispecies. The determination of diversity and complexity showed that the mutation rate and complexity of HY12 virus quasispecies increased, while the proportion of HY12 VP1 and VP2 consensus (master) sequences decreased with increasing passages. Synonymous mutation and non-synonymous mutation analysis displayed a positive selection for HY12 quasispecies evolution. A comparison of HY12 virus in different passages demonstrated that HY12 virus altered its characteristic, phenotype, and pathogenicity via non-synonymous mutation. These findings revealed the evolution pattern for HY12 virus, and the alteration of HY12 virus characteristics and pathogenicity by mutation.

## 1. Introduction

Virus uses different strategies to survive the changeable environments. Enterovirus, like the majority of RNA viruses, employs the strategies of mutation to adapt the surroundings and implicate in pathogenesis [1,2,3,4]. As an important causative agent, enterovirus is a member of the genus of *Enterovirus* within the family of *Picornaviridae*. According to the latest virus classification by International Committee on Taxonomy of Viruses, the genus of *Enterovirus* consists of 12 species of enterovirus and 3 species of rhinovirus [5,6]. Out of 12 enterovirus species, EV-A, EV-B, EV-C, and EV-D are the causative agents of human diseases such as poliomyelitis, hand, foot, and mouth disease (HFMD), and coxsackievirus infection [7,8,9], while EV-E and EV-F are the causative agents of bovine enterovirus infection characterized by digestive, respiratory, and neurological disorders, and even subclinical infection [10,11,12,13].

The genome of enterovirus contains a single open reading frame (ORF) flanked by 5′-UTR and 3′-UTR [14]. The ORF encodes a precursor polyprotein of approximately 2176 amino acids, which is subsequently processed by viral or cellular proteases into 11 individual proteins including VP4, VP2, VP3, VP1, 2A, 2B, 2C, 3A, 3B, 3C, and 3D from the N terminus to the C terminus [15,16]. VP1, VP2, VP3, and VP4 are four structural proteins, and the rest are nonstructural viral proteins. VP1 was demonstrated to play a vital role in virus entry, viral pathogenicity, and virus elicited immunity [17,18,19,20]. Similar to other bovine enteroviruses (BEV), the complete genome sequence of EV-E HY12 strain is composed of 7469 nucleotides, where it contains one ORF encoding a polyprotein of 2176 aa, a 5′-UTR consisting of 817 nucleotides, and 3′-UTR containing 68 nucleotides followed by a poly (A) tail with 53 polyadenines. Alignment analysis revealed that HY12-encoded VP1 shares eight variable regions and six highly or relatively conserved regions with other bovine enteroviruses [21].

Quasispecies theory was developed in the 1970s by Manfred Eigen and Peter Schuster to explain self-organization and adaptability of primitive replicons that link genotypic and phenotypic information in the origin of life [22,23]. Virus quasispecies refers to a virus population composed of a series of virus mutants with similar but different gene sequences. These mutants continue to experience genetic mutation, competition, and selection in response to a variety of external factors, which is the driving force for virus evolution. In an infected cell, the virus quasispecies is considered as a single replication unit, and produced by both mutation and recombination [24,25].

Virus mutation occurs during viral replication. Since RNA-dependent RNA polymerase (RdRP) encoded by RNA virus has low fidelity, mutants are easily generated during virus replication [26,27,28], which provides a prerequisite for virus evolution. Mutation is divided into synonymous and non-synonymous mutations (dN-dS). Synonymous mutation has no change of amino acid sequence when a nucleotide change occurs. This type of mutation was intuitively thought to be functionally silent or evolutionarily neutral. However, recent studies showed that optimized synonymous codon usage is beneficial to the speedy mRNA translation [29,30]. In comparison to synonymous mutation, the nonsynonymous mutation changed the amino acid sequence, altered the function, and were frequently subjected to natural selection. The ratio of dN-dS is usually used as a criterion for selection. When dN > dS, it is positive selection, a process in which genotypes are dominant in the evolutionary population and which is conducive to heterogeneous mutation. In contrast, when dN < dS, it is negative selection, and the genotype is cleared in an evolutionary population. This type selection is not conducive to non-synonymous mutation and will not be beneficial to virus growth and survival [31,32,33].

Viral mutants persisting in the population can change the tissue tropism, host range, and virus virulence [34,35]. It has been demonstrated that high mutation rate plays an important role in virus survival, pathogenesis, and adaption to the complex environment. During the process of enterovirus infection, moderate genetic mutation occurs rapidly and alters the virus virulence [36]. It was reported that mutation in 5′UTR and the VP1 gene of poliovirus changes its neurotoxicity to a great extent. Deletion in the regulatory region of the poliovirus genome reduces virus virulence [36]. Mutation of a single amino acid in VP1 protein for EV71 is related to the neurotoxicity and recognition of receptor [37]. Early studies also showed that enterovirus causes persistent infection in specific tissues or cells after it infects the body, and the virus is co-evolved in persistently infected cells [38].

Bovine enterovirus infection manifests several clinical signs as such digestive, respiratory, reproductive, and neurological disorders in addition to subclinical infection. Previous studies revealed the genetic and biological variations of BEV strains isolated from cattle manifesting different clinical signs and demonstrated that some BEV strains caused severe digestive and respiratory disease, while other strains caused only mild infection [21,39,40]. Elucidation of the underlying mechanism and insight into the evolution of the virulence and pathogenicity of BEV will provide the prerequisite for prevention of this infection. In this study, we used the HY12 virus as a model to determine the evolution pattern of virus virulence and quasispecies in an attempt to address our hypothesis that the mutation of enterovirus leads to virus virulence alteration by analyzing viral structural protein VP1 and VP2 sequence from different passages. We found that HY12 enterovirus exists as an array of closely related mutants, revealed the evolution pattern of quasispecies, and demonstrated that HY12 virus alters its phenotype, characteristics, and pathogenicity via non-synonymous mutation, thus likely affecting the outcome of clinical modes.

## 2. Materials and Methods

### 2.1. Ethics Statement

The procedures used for this study, including those involving mice, followed a standard protocol reviewed and approved by the Institutional Animal Care and Use Committee (IACUC) of Jilin University (approval no JLU-20150226), following strict compliance with the requirements of the Animal Ethics Procedures and Guidelines of the People’s Republic of China. Pregnant ICR mice were obtained from Changchun Biological Products Institute. The mice were maintained in the Laboratory Animal Facility of Jilin Province. All mice had free access to food and water and were kept in a temperature-controlled room (22 ± 0.5 °C) on reverse 12/12 h light/dark cycle. The new-born pups aged three days were randomly assigned to different treatment groups with each cage litter containing a dam and 5–10 pups.

### 2.2. Cell Culture

Africa green monkey kidney cells were cultured in Dulbecco’s modified Eagle’s medium (DMEM) (Invitrogen, Carlsbad, CA, USA), supplemented with 10% fetal bovine serum (HyClone, Beijing, China), 2 µg/mL gentamycin, and 2 mM l-glutamine in 5% CO_2_ at 37 °C. After infected by virus, the cells were maintained with DMEM containing 2% FBS, 2 µg/mL gentamycin, and 2 mM l-glutamine.

### 2.3. HY12 Virus and Its Serial Passages in Vero Cells

HY12 virus is an enterovirus isolated from cattle herd manifesting severe diarrhea and respiratory signs [21]. After titration, HY12 virus was continuously passaged in Vero cells for 120 generations. The cells infected using 100 TCID_50_ of HY12 virus for each generation were harvested and stored at −80 °C for future use.

### 2.4. Virus Titration and Plaque Assay

TCID_50_ was determined following a normal procedure and calculated according to the Reed–Muench method [41]. Plaque assay was performed following a standard method [42]. A representative passage HY12 virus was 10× serially-diluted with DMEM and used as virus inoculum. 100 μL of diluted inoculum was added to each well in a 6-well plate with a triple repeat. After incubation at 37 °C for 2 h, the cells were washed twice with Hank’s balanced salt solution before adding 2 mL of 0.8% low melting point agarose. After plaque grew to the visible size, the agarose layer was removed. Cells were fixed with cold methanol at −20 °C for 30 min and stained with crystal violet for 1–2 h. The resulting plaques were measured and photographed using a Nikon digital camera.

### 2.5. RNA Extraction, cDNA Synthesis, and Amplification of 5′-UTR, VP1, and VP2 Gene Sequences

Total RNAs were extracted from cells infected by representative passages of HY12 virus (P2, P20, P40, P60, P80, and P100) following procedures as previously described [21]. cDNA synthesis was carried out using the Bio RT-cDNA kit (Invitrogen) following the manufacturer’s instruction. The cDNA was synthesized at 42 °C for 1 h before storage at −20 °C.

Amplification of the 5′UTR (P2), VP1, and VP2 genes from representative passage of HY12 (P2, P20, P40, P60, P80, and P100) was performed by using PCR kit (Takara, Dalian, China) following the manufacturer’s instruction. Primers for amplification of the 5′-UTR, VP1, and VP2 genes were listed as follows. HY12-VP1-S: 5′-GCGTTGACGATACCTTAAACTA-3′, HY12-VP1-AS: 5′-CTCTGATATGACTGCCATACCT-3′; HY12-VP2-S: 5′-ATCAGCCGCCCAGAACAAGCA-3′, HY12-VP2-AS: 5′-TGTTGGTTGGAAATCAGGAAGAATG-3′; HY12-5′-UTR-S: 5′-CCTTTGTACGCCTGTTTTCCCCACC-3′, HY12-5′-UTR-AS: 5′-ACACGCCCGGAGGTTAGGATTAGCA-3′. PCR-amplified 5′-UTR, VP1, and VP2 genes were either directly sent out for sequencing or cloned to pEGM^®^-T vector (Promega, Madison, WI, USA) before sequencing (Sangon Biotechnology, Shanghai, China).

### 2.6. Sequence Alignment Analysis

Alignment analysis was performed using Clustal W methods [43]. VP1 and VP2 sequences from representative passage of HY12 virus were aligned and analyzed in the clones from the same passage or the clones from the different passages.

### 2.7. Investigation on the Diversity and Complexity for HY12 Virus Quasispecies

The diversity for HY12 quasispecies was determined following the methods described previously [44]. A total of 20 clones in representative passages were selected and sequenced. Sequences from 20 clones in the same passage or different passages were then compared and analyzed to determine the number of mutation sites. The complexity of HY12 virus quasispecies was expressed as the ratio of the number of clonotypes to total number of clones. The mutation rate, complexity, and the proportion of the main sequence in the quasispecies and the homology of nucleotides between different clones were analyzed for HY12 enterovirus as described previously [33].

### 2.8. Analysis of the Evolution for HY12 Virus Quasispecies

Evolution for HY12 virus quasispecies was analyzed using the methods previously described [45]. Synonymous mutations and non-synonymous mutations were performed using online analysis software (http://www.datamonkey.org (accessed on 24 September 2021)) to analyze the ratio of dN/dS for VP1 and VP2 genes. The ratio of synonymous and non-synonymous mutations is used as a criterion for natural selection, where it is considered as positive selection when dN/dS is > 1.0, and negative selection when dN/dS is <1.0.

### 2.9. Pathogenicity Comparison for HY12 Virus in Representative Passages

Pathogenicity for P2 and P40 HY12 virus was comparably determined using the established neonatal mouse model as described [46]. Three groups of neonatal ICR mice at the age of 3 days old were intraperitoneally inoculated with 10^8^ TCID_50_ of P2 and P40 HY12 virus. Control neonatal mice were injected with an equal amount of DMEM medium. Mice were observed every 4–6 h. Lung, intestine, and brain tissues were collected in 14 dpi after mice were euthanized.

### 2.10. Tissue Processing and Microscopic Examination

Tissues collected from mice infected with P2 and P40 HY12 virus were processed in the procedure described previously [46]. Paraffin-embedded tissue blocks were sectioned at 5 μm using a microtome. The ribbon sections were loaded to polylysine-coated glass slides, dried overnight at 42 °C, and stained using hematoxylin and eosin (H&E) staining as previously described [46]. Microscopic lesions were visualized and captured using a CCD camera mounted on a Nikon epifluorescence microscope (Nikon Instruments Co., Ltd., Shanghai, China).

### 2.11. Quantitative Real-Time PCR

Quantitation of viral genome copies in cells infected by P2 and P40 HY12 virus was performed using quantitative real-time PCR following the manufacturer’s instruction. The primers used for real-time PCR were listed as follows. HY12-U: 5′-CAATAACTACTACCCAGAACGGATG-3′; HY12-D: 5′-GTCAGCGAACCCAACAAGATTA-3′. GAPDH was used as calibration control, the primers were listed as follows. GAPDH-U: 5′-GTCTTCACTACCATGGAGAAGG-3′; GAPDH-D: 5′-TCATGGATGACCTTGGCCAG-3′.

### 2.12. Statistical Analysis

Statistical analyses were performed using SPSS Statistics software. TCID_50_ titer, mRNA, and protein expressions were statistically analyzed by the one-way ANOVA test. The results were expressed as means ± standard deviation of the means (SD) from three independent experiments. Results were considered extremely significant when the *p* value was less than 0.01.

## 3. Results

### 3.1. Homogeneity of HY12 Virus in Early Passages

**Figure 1:**
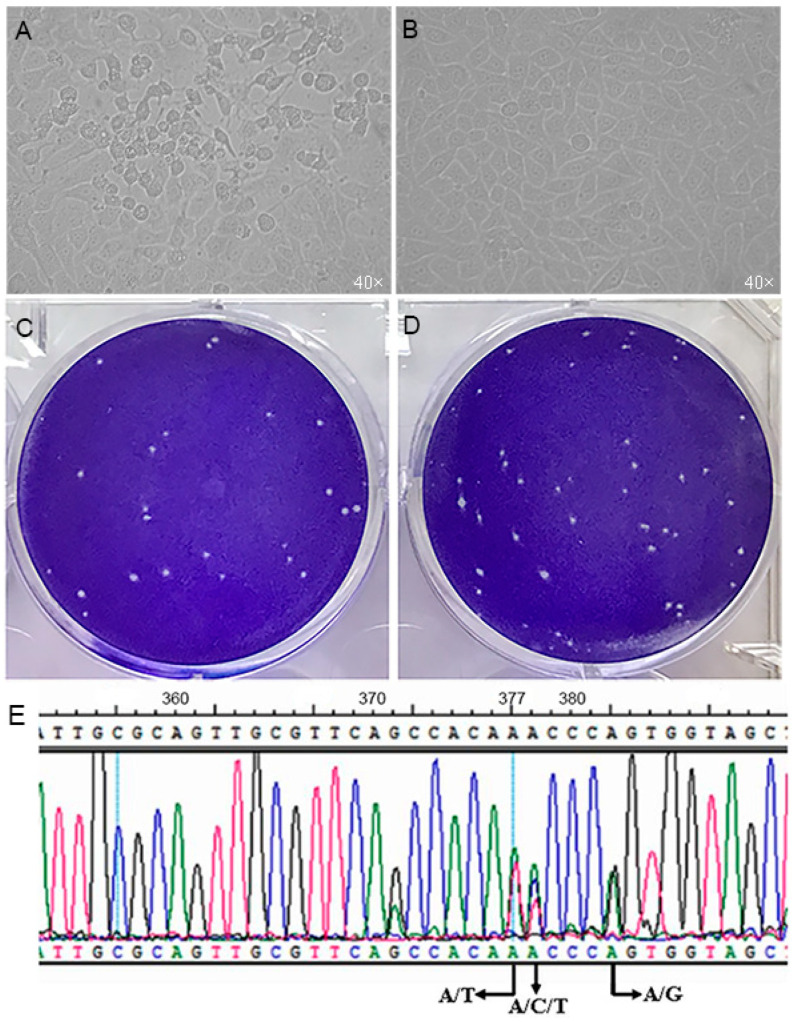
Property characterization for early passage HY12 enterovirus. (**A**), cytopathic effect observed in Vero cells infected by P2 HY12 virus in comparison with normal Vero cell (**B**). (**C**,**D**), plaque assay showed a relative homogeneity of plaque size for P2 HY12 virus. (**E**), unveiling of HY12 virus as an array of mutants (quasispecies). 5′UTR region for P2 HY12 virus was amplified and sequenced. Two or three nucleotides overlapped in the same nucleotide positions revealed like A/T bases at the position 377, A/C/T bases at the position 378, and A/G bases at the position 382 in relation to HY12 virus complete genome sequence.

First, 100 TCID_50_ of HY12 virus (P2) was used to infect the Vero cells cultured in a 60 mm plate. After incubation with the inoculum, Vero cells showed a typical cytopathic effect as early as 6–8 hpi, became rounded, and detached off the flask in 24–48 hpi (Figure 1A) in comparison with the normal Vero cell control (Figure 1B). The TCID_50_ titer was determined as 10^−9^/0.1 mL (data not shown). The plaque assay showed a similar morphology with a middle-sized plaque for HY12 virus, indicating the homogeneity of HY12 virus in early passages (Figure 1C,D).

### 3.2. An Array of HY12 Virus Mutants Revealed by Direct Sequencing PCR-Amplified 5′-UTR Region

In the course of unveiling the complete genome sequence for HY12 virus, a pair of primers were designed and used to determine the 5′UTR sequence. After the PCR-amplified 5′UTR fragment was directly sequenced, it was surprising to note that multiple nucleotides were observed in the same nucleotide positions including A/T bases at the position 377, A/C/T bases at the position 378, and A/G bases at the position 382 in relation to the HY12 virus complete genome sequence (Figure 1E), suggesting that the PCR-amplified product is a mixture of fragments with different mutations. In other words, the HY12 enterovirus is a virus population containing an array of similar but different mutants, the quasispecies.

### 3.3. Diversity and Complexity of HY12 Quasispecies

Since the HY12 virus was revealed as a quasispecies, it was interesting to investigate the diversity and complexity of HY12 quasispecies. VP1 and VP2 genes were chosen for PCR amplification since they are viral structure proteins, involved in virus infection, and elicited immunity as well as being relatively variable based on the sequence analysis. After PCR-amplified VP1 and VP2 fragments from P2 HY12 virus were cloned into pEGM^®^-T vector, 20 clones for VP1 and VP2 were randomly picked and processed for sequencing. Sequence analysis showed that out of 20 VP1 gene clones, 4 clones had nucleotide mutations in different sites, while 16 clones had the identical gene sequences to the original sequence of HY12 (Table 1). Similarly, out of 20 VP2 gene clones, 6 clones had different mutation sites in comparison to 15 clones with the identical sequence to original HY12 virus (Table 2). These results demonstrate the diversity of HY12 virus quasispecies.

To determine the complexity of HY12 virus quasispecies, the clonotypes and ratio of clonotypes to total clones were determined and analyzed. As shown in Table 1, 4 nucleotide mutation sites were revealed in VP1 gene for P2 HY12 virus, which led to 4 clonotypes with a 20% of quasispecies complexity. Similarly, as illustrated in Table 2, 6 nucleotide mutation sites were revealed in VP2 gene of P2 HY12 virus, leading to 4 clonotypes with a 20% quasispecies complexity. These results demonstrate the complexity of HY12 quasispecies.

### 3.4. Phenotype Alteration for HY12 Quasispecies

**Figure 2:**
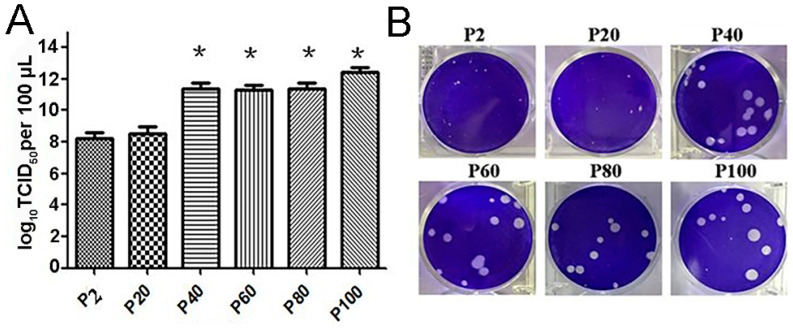
Alteration of phenotype of HY12 virus with increasing passages. (**A**), TCID_50_ titer increased with the increase of virus passages in Vero cells. TCID_50_ titer was assayed for a representative passage of HY12 virus (P2, P20, P40, P60, and P100) and represented as log 10/0.1 mL (* *p* < 0.05). (**B**), Alteration of plaque size for HY12 Vero cells. Plaque sizes were observed to increase approximately 30–40 times after HY12 virus passage in Vero cells for 40 and over 40 generations (P40, P60, P80, and P100) in comparison with those of P2 and P20 virus.

To explore whether HY12 quasispecies experience any evolution and evolution pattern, P2 HY12 virus was continuously passaged in Vero cells until P120 HY12 virus was harvested. Each passage of HY12 virus was titrated and used as inoculum to infect Vero cells. Representative passages of HY12 virus (P2, P20, P40, P60, P80, P100) were selected to compare their characteristics as such TCID_50_ and plaque size. As shown in Figure 2A, TCID_50_ titer for P20 virus was similar to P2 virus, about 10^−9^/0.1 mL, while TCID_50_ titers for P40, P60, P80, and P100 virus (10^−11^/0.1 mL–10^−12^/0.1 mL) were significantly higher than that of P2 virus, indicating that HY12 virus titer significantly increased when the virus was passaged in Vero cells over 40 generations. Similarly, the size of plaque for P20 virus was relatively small, similar to the size for P2 virus. However, it was surprising to note that the sizes of plaque for the representative passages (P40, P60, P80, and P100) virus were relatively homogeneous and increased to 30–40 times larger in relation to that in P2 virus (B). These results demonstrated the alteration of HY12 virus characteristic in the course of its passage in cell culture.

### 3.5. Mutation Sites in VP1 and VP2 Gene for HY12 Virus

**Figure 3:**
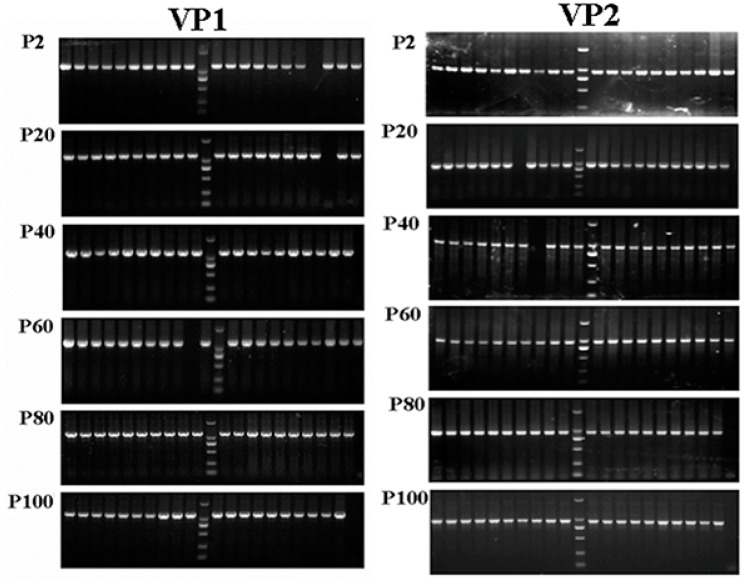
VP1 and VP2 genes were amplified from representative passages of HY12 virus. VP1 and VP2 gene fragments were amplified from transformed bacterial colonies using single colony PCR. A total of 20 colonies were selected from P2, P20, P40, P60, P80, P100 virus. Expected size of fragments for VP1 and VP2 were obtained from 20 clones.

To explore the potential genetic variations responsible for the alteration of HY12 virus phenotype changes, VP1 and VP2 genes were selected for further investigation since they play a crucial role for virus entry and elicited immunity. VP1 and VP2 genes for P2, P20, P40, P60, P80, and P100 virus were amplified by RT-PCR. After the fragments were cloned to the pGEM^®^-T vector, 20 clones for each representative passage were randomly selected, amplified by single colony PCR (Figure 3), and sequenced. Sequence analyses revealed mutations in VP1 genes in different passages (Table 3). Out of the 20 clones for P2 virus, 2 clones had a synonymous mutation at nucleotide position 72, changing from adenine to guanine (72 A > G). However, this mutation site was not stably inherited in the subsequent passage. With the passage number increased to or over 40, several new mutation sites appeared. Out of 20 clones of P40, 19 clones had a mutation of guanine at position 272 to adenine (272 G > A), resulting in mutation of amino acid from arginine to histidine (R91H). This non-synonymous mutation was still observed in 19/20 clones in P60 virus, and all 20 clones for P80 and P100 virus with a 100% mutation proportion. In addition, a mutation at nucleotide position 456 was also observed. Out of 20 clones, 16 clones had a mutation at position 456 from guanine to adenine (456 G > A). However, this point of mutation did not cause the change of amino acid. Sequencing the VP1 gene from P60 to P100 virus revealed that all clones had the same mutation at 456 (G > A), with a mutation proportion of 100%. Simultaneously, a synonymous mutation of 723 T > C was found in P80 virus with a mutation proportion of 60%. This mutation reverted to its original status in P100 virus (723C > T). Furthermore, two new synonymous mutation sites in P100 passage were revealed including 609 T > C and 652 C > T, with mutation rates of 15% and 25%, respectively.

Compared with the VP1 gene, the VP2 gene was relatively well conserved during virus passage. As shown in Table 4, two regular mutation sites were observed in the VP2 gene after virus passage reached P100. Mutation of cytosine to guanine at position 442 (442 C > G) resulted in the change of amino acid from glutamine to glutamic acid (Q148E) with a mutation proportion of 75%. Mutation of thymine to cytosine at position 667 (667 T > C) was a synonymous mutation with 10% mutation proportion.

### 3.6. Evolution Pattern for HY12 Quasispecies

To unveil the evolution pattern for HY12 virus quasispecies, the master sequence, proportion of dominant quasispecies, complexity, the number of mutation sites, mutation rate, and homology analysis for P2, P20, P40, P60, P80, and P100 virus were analyzed. As shown in Table 1, the master sequence (dominant quasispecies) of VP1 gene changed with the increasing passage number. Proportion of the original master sequence gradually decreased from 80% for P2 virus to 35% for P100 virus. When HY12 virus was passed to the P40 generations, the original master sequence disappeared and a new master sequence evolved. The proportion of the new master sequence in the quasispecies gradually decreased as the passage number increased. Simultaneously, the number of clonotypes increased, from 4 clonotypes for P2 virus to 12 clonotypes for P100 virus, resulting in the increase of complexity for HY12 quasispecies from 20% in P2 generation to 60% for P100 generation. Diversity for HY12 quasispecies was also determined and shown in Table 1. The number of nucleotide mutation sites increased from 4 in P2 generation to 69 in P100 generation in the process of virus passage, resulting in the increase of mutation rate from 2.36 × 10^−4^ gradually to 40.78 × 10^−4^.

Compared with the VP1 gene, the variation in the VP2 gene was small. As shown in Table 2, the proportion of master sequence for VP2 gene decreased slowly during passage and a new master sequence did not evolve until in P100 generation. Like the observation in VP1 gene, the clonotypes in VP2 also increased with the increasing passages, from 4 in P2 virus to 14 in P100 generation. The complexity of quasispecies increased from 20% in P2 generations to 70% in P100 generations. The proportion of dominant quasispecies decreased gradually, from 75% in P2 generations to 30% in P100 generations. The number of nucleotide mutation sites and mutation rate increased gradually with the increase of the passage times.

Taken together, the above results demonstrated that the mutation rate and complexity of HY12 virus quasispecies increased, and the proportion of HY12 VP1 and VP2 consensus (master) sequences decreased with the increasing passage.

### 3.7. HY12 Virus Quasispecies Evolved in a Positive Selection

**Figure 4:**
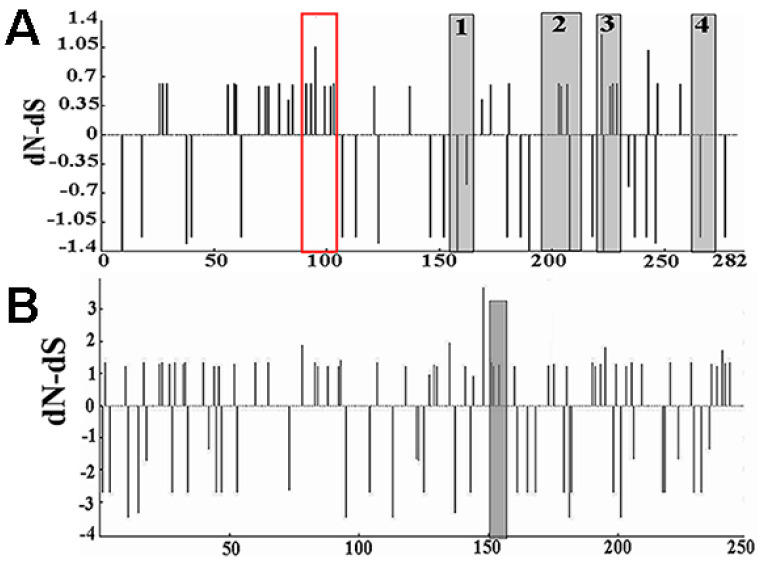
Analysis of selection pressure on VP1 gene and VP2 gene. (**A**) Natural selection pressure on VP1 protein. Four antigenic epitope regions in VP1 protein were revealed named regions 1, 2, 3, and 4 in addition to the receptor binding region (marked as red box). Negative selection was found in region 1 and region 4 (dN < dS). Positive selection was found in region 2, region 3, and receptor binding region, suggesting they were conducive to the virus adapting to the environment. (**B**) Prediction on natural selection pressure on VP2 protein. The marked area is the epitope area in VP2. dN > dS was revealed in this region, indicating that it is beneficial for virus evolution.

To determine whether any regions in VP1 and VP2 genes bear external pressure, analysis software (http://www.datamonkey.org/dataupload.php (accessed on 24 September 2021)) was applied to conduct dN-dS analysis on VP1 and VP2 genes, respectively. Selection pressure on VP1 and VP2 proteins was evaluated according to the value of dN-dS. As shown in Figure 4A, four antigenic epitope regions (1, 2, 3, and 4) in VP1 were detected with their corresponding amino acid sequences as follows: PADQDSYQWQ, YARFMNTDPDKYGILPSNF, RFRIYAKIKH, and RYHLVFGGPNFQDKICADRA, in addition to the receptor binding region. In region 1 and region 4, the two antigenic sites were relatively conserved. The synonymous mutations in these regions were larger than the non-synonymous mutations (dN < dS), suggesting that the selection was a negative selection and these two regions were not conducive to the evolution of the virus under the selection pressure. Compared with region 1 and 4, the non-synonymous mutations in region 2 and region 3 were larger than the synonymous mutations (dN > dS), indicating the selection was a positive selection and it was conducive to virus evolution. The dN > dS revealed in amino acid region of receptor binding (70–110) within VP1 protein indicated that this region was more prone to non-synonymous mutations, which is beneficial for the virus to better adapt to the external environment changes.

Analysis of dN-dS in VP2 protein revealed an antigen epitope region marked with amino acid sequence of FQEAFWLEDG (Figure 4B). The dN > dS in this region demonstrated that non-synonymous mutation was greater than the synonymous mutation, which was a positive selection and beneficial to the virus evolution.

### 3.8. Alteration of the Dominant HY12 Virus Quasispecies

**Figure 5:**
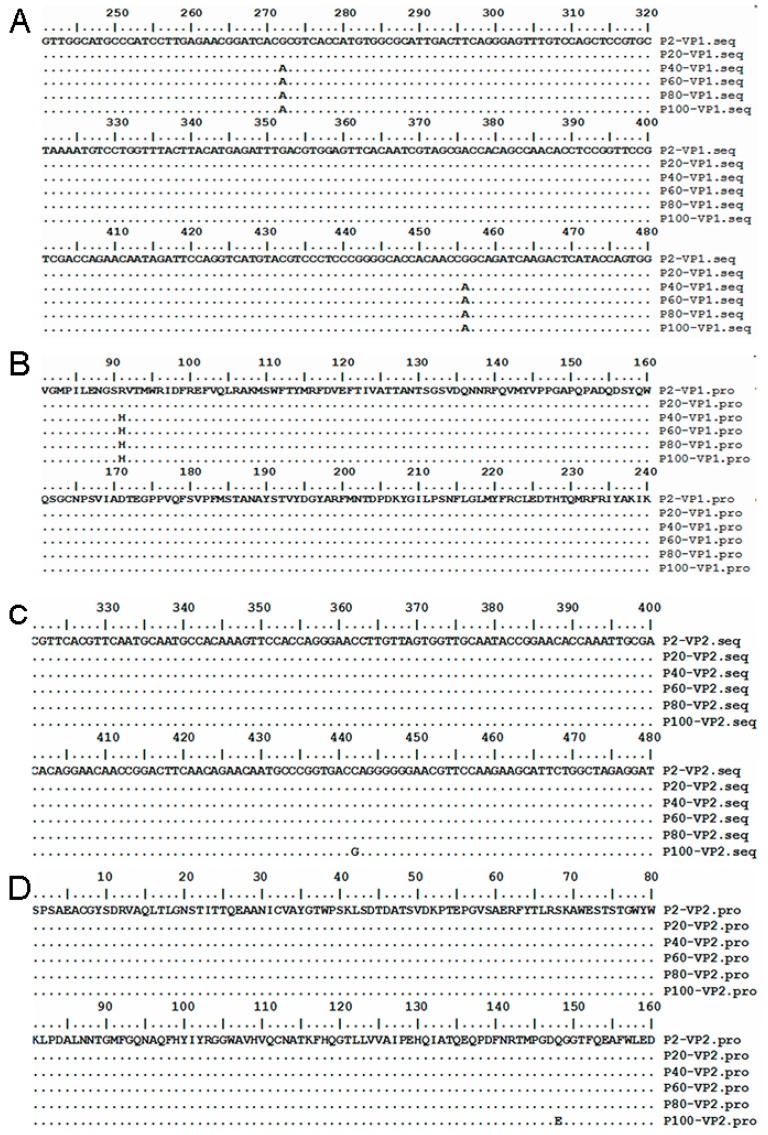
Mutation of VP1 and VP2 genes for HY12 virus in representative passages. (**A**) Nucleotide sequence mutation revealed in VP1 gene. VP1 genes were amplified and sequenced from cells infected with representative passage of HY12 viruses. Two mutations were revealed in HY12 viruses over 40 passages, with one non-synonymous mutation located at position 272G > A of VP1 gene, and another synonymous mutation at position of 456G > A. (**B**) Amino acid mutation in VP1 gene. An amino acid mutation from arginine to histidine was found (R91H). (**C**) Nucleotide sequence mutation in VP2 gene. Nucleotide mutation at position 442 (442 C > G) was found in P100 virus. (**D**) the amino acid mutation in VP2. Mutation of glutamine to glutamic acid (Q148E) at position 148 was revealed.

Analyses of the mutation sites in VP1 and VP2 gene from different generations for HY12 virus quasispecies revealed the change of the master sequences in different generations. As shown in Figure 5A,B, the master sequence of VP1 did not change when HY12 virus was passed to P20 generations. However, it changed in P40 to P100 generations due to the mutation of guanine to adenine (272G > A) at position 272 and 456. Mutation of nucleotide at position 272 belonged to non-synonymous mutation and resulted in the alteration of amino acid from arginine to histidine at position 91(R91H), which was persistently present as the dominant mutant in or over P40 generations. The nucleotide mutation at position 456 was a synonymous mutation although it also presents in or over P40 generation. As illustrated in Figure 5C,D, the mutation of the nucleotide at position 442 (442 C > G) in VP2 was revealed just in P100 generation, resulting in a mutation of amino acid from glutamine to glutamate at position 148 (Q148E) in VP2 protein.

### 3.9. Mutation of R91H in VP1 Protein Leads to Secondary and Tertiary Structure Alteration

**Figure 6:**
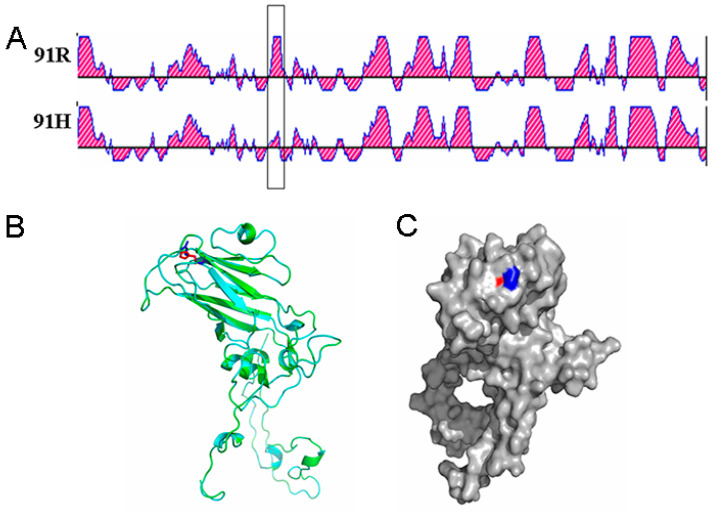
Alteration of VP1 antigenicity and tertiary structure by mutation of R91H. (**A**) Effect of R91H mutation on VP1 antigenicity. VP1 antigenicity was predicted using the Lasergene software for VP1 gene with or without R91H mutation. Antigenicity of VP1 with R91H was altered in relation to VP1 protein deduced from parental HY12. (**B**,**C**) Amino acid position shift in VP1 tertiary structure analyzed using Pymol software. Blue spot refers to the amino acid residue arginine, the red spot stands for histidine amino acid residue.

To determine whether R91H mutation had any effect on VP1 structure, antigenicity, or function, secondary structure was predicted using Lasergene software. As shown in Figure 6A, mutation of R91H in VP1 indeed changed the antigenicity of VP1 protein greatly. To examine if the mutation of R91H had any effect on its tertiary structure, Phymol software was used to predict and analyze VP1 tertiary structure. As shown in Figure 6B,C, R91H mutation in VP1 protein caused the shift of the amino acid at this site in the tertiary structure. The amino acid residue R (blue color) outside of the “canyon” mutated to H residing at the inside of canyon (the red color).

### 3.10. R91H Mutation Alters the Replication Efficiency and Pathogenicity for HY12 Virus Quasispecies

**Figure 7:**
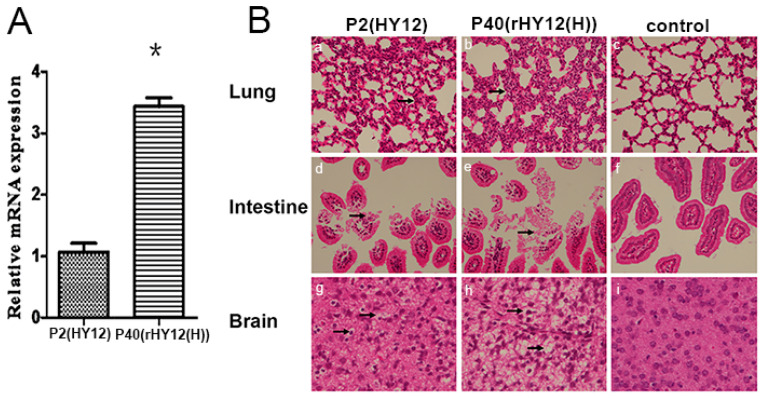
Replication and pathogenicity were enhanced by mutation of R91H. (**A**) VP2 mRNA expression was detected and compared in Vero cells infected by P2 and P40 HY12 virus (* *p* < 0.05). (**B**) Histopathological changes on lungs, intestines, and brains from mice infected by P2 and P40 HY12 virus. Mice injected with equal amounts of DMEM were used as control. a and b were the pathological changes in lung; d and e represent the pathological changes in intestine; g and h were the pathological changes in brain; c, f, and i were the controls for lung, intestine, and brain. Arrow: histopathological changes.

To compare the difference of replication for P2 and P40 virus, qPCR was used to quantitate the viral transcript mRNA level in Vero cells infected by equal amount (100 TCID_50_) of P2 and P40 HY12 virus. As shown in Figure 7A, the VP2 transcript level in cells infected by P40 HY12 virus was significantly higher in relation to that in cells infected by P2 virus (*p* < 0.05), indicating that the mutation R91H in VP1 protein significantly enhanced the efficiency of virus replication.

To explore whether the R91H mutation has any influence on viral pathogenicity, suckling mice were infected by P2 and P40 virus. Clinical signs and histopathological changes of suckling mice in different infection groups were observed. Suckling mice infected by P40 virus showed much more severe signs such as trembling and growth retardation than those infected by P2 HY12 virus. Histopathological examination revealed much more severe histopathological changes in the lung, brain, and intestine tissues from mice infected by P40 virus (Figure 7B), including serious alveolar collapse and thickening of alveolar wall in lung, obvious swelling, degeneration, and severe shedding of intestinal villi for the intestinal mucosal epithelial cells of duodenum, and severe vacuolar degeneration and diffuse inflammatory cell infiltration in brain cells. These results suggest that mutation of R91H affected the replication and pathogenicity for HY12 virus in the course of its evolution.

## 4. Discussion

In this study, we employed the HY12 enterovirus to explore its evolution and the underlying mechanism in a cell model system. We discovered the existence of HY12 virus in a form of quasispecies, unveiled the evolution pattern for the complexity, diversity, and mutation pattern for HY12 virus quasispecies, and revealed the alteration of HY12 characteristics, phenotype, and pathogenicity via non-synonymous mutation.

Mutation is one of the strategies widely used by viruses for their fitness and survival. RNA viruses exploit this strategy to generate the closely related diverse mutants called quasispecies to encounter the changeable environment and host defense system [4,47]. We employed the cell model system to serially passage HY12 virus and compare the characteristics and phenotype to explore virus evolution and pattern. By direct sequencing and analyzing the PCR-amplified 5′UTR fragment from P2 virus, we surprisingly found two or three nucleotide sequences were overlapped in several positions such as A/T bases at the position 377, A/C/T bases at the position 378, and A/G bases at the position 382 in relation to the HY12 virus complete genome sequence. These findings indicate that the PCR-amplified product was actually a mixture of fragments with different mutations, thus demonstrating that HY12 virus exists as an array of closely related mutants, the quasispecies.

VP1 and VP2 genes were further selected for PCR amplification and sequencing since they are viral structural proteins involved in virus infection and immune evasion from host defense. Sequence analysis on the amplified VP1 and VP2 genes from P2 virus also revealed the existence of different mutants and clonotypes, suggesting the diversity and complexity of HY12 virus quasispecies. Quasispecies refer to the virus population composed of different closely related mutants. These mutants continue to undergo genetic variation and competitive selection in the process of evolution. The characterization of the HY12 virus in different passages showed a dramatic phenotype change, where plaque size of HY12 virus in P40 or over P40 generations was 30–40 times larger than that in P2 virus. Simultaneously, the TCID_50_ titer was significantly increased in P40 or over P40 passages. These results demonstrated the alteration of properties of HY12 virus. Analyses on the master sequence, the proportion of dominant quasispecies, the complexity, the number of mutation sites and mutation rates, and homology for P2, P20, P40, P60, P80, and P100 virus revealed the HY12 quasispecies evolution and evolution pattern. With the increase of the passages, the diversity of HY12 quasispecies increased, while the mutation rates and the complexity for HY12 quasispecies decreased. These results were consistent with the evolution pattern for many RNA viruses and were likely beneficial for HY12 virus survival [1]. As a driving force for evolution, mutations revealed for HY12 virus in the progress of virus passages are likely the mechanism for the virus adapt to different environments and produce high adaptability, which is also evidenced by the positive natural selection revealed for the VP1 protein in this study.

Mutation of the VP1 gene was demonstrated to change virus binding ability to cells, affect viral infectivity, virulence, and pathogenicity [47]. Comparison analysis on VP1 gene from different generations showed several consistent mutations for HY12 virus passaged over 40 generations. One mutation in the VP1 gene was 272G > A, leading to a non-synonymous mutation of R91H. Another mutation was 456G > A, a synonymous mutation. Interestingly, the non-synonymous mutation of R91H in VP1 was predicted to have an effect on its secondary and tertiary structures, suggesting that the mutation (R91H) is likely related to characteristic alterations for HY12 virus observed in its passages in Vero cells. To confirm the above supposition, we generated the HY12 cDNA infectious clone and used reverse genetic approaches to obtain HY12 viruses possessing the mutation of R91H and discovered that TCID_50_ and the plaque size of rescued HY12(H) were significantly increased in comparison to those of rescued wild-type HY12 virus, demonstrating that R91H mutation is truly responsible for the alteration of HY12 infectivity and the increase of plaque size (data not shown).

VP1 is the most variable structural protein for enteroviruses. In relation to other picornaviruses, the capsid protein VP1 of BEV contains five regions lying at or within the so-called canyon (R1: aa 86–92, R2: aa 127–138, R3: aa 198–208, R4: aa 226–230, and R5: aa 266–273) [48]. These five regions were shown to be involved in capsid protein–host receptor binding. Our findings that non-synonymous mutations in region 2 and region 3 is larger than the synonymous mutations (dN > dS) indicate that positive selection is conducive to HY12 virus evolution. Interestingly, R91H mutation and dN > dS were shown within 70–110 aa of VP1 protein, indicating that this region was more prone to non-synonymous mutations and beneficial for HY12 virus to better adapt to the external environment changes. Although R91H mutation of HY12 virus was found located in R1 region, whether this mutation could affect the binding of HY12 virus to its receptor remains unclear, which is a subject for future investigation.

## Figures and Tables

**Table 1:** Analysis of variation pattern in different passages VP1 gene of HY12.

Different Generations	Total Number of Clones	The Number of Clones Identical to the Original Master Sequence	Main Sequence Clone Number	Proportion of Dominant Quasispecies	Number of Clonotypes	Quasispecies Complexity	Nucleotide Mutation Sites	Mutation Rate	Nucleotide Homology
P2	20	16	16	80%	4	20%	4	2.36 × 10^−4^	99.8%–100%
P20	20	14	14	70%	6	30%	6	3.55 × 10^−4^	99.7%–100%
P40	20	0	13	65%	6	30%	38	22.46 × 10^−4^	99.5%–100%
P60	20	0	11	55%	10	50%	49	28.96 × 10^−4^	99.5%–100%
P80	20	0	7	35%	14	70%	72	42.55 × 10^−4^	99.5%–100%
P100	20	0	7	35%	12	60%	69	40.78 × 10^−4^	99.3%–100%

**Table 2:** Analysis of variation pattern in different passages VP2 gene of HY12.

Different Generations	Total Number of Clones	The Number of Clones Identical to the Original Master Sequence	Main Sequence Clone Number	Number of Clonotypes	Proportion of Dominant Quasispecies	Quasispecies Complexity	Nucleotide Mutation Sites	Mutation Rate	Nucleotide Homology
P2	20	15	15	4	75%	20%	6	4.03 × 10^−4^	99.8%–100%
P20	20	14	14	6	70%	30%	6	4.03 × 10^−4^	99.7%–100%
P40	20	13	13	8	65%	40%	7	4.73 × 10^−4^	99.7%–100%
P60	20	7	7	14	35%	70%	20	13.45 × 10^−4^	99.6%–100%
P80	20	6	6	14	30%	70%	21	14.11 × 10^−4^	99.6%–100%
P100	20	0	6	14	30%	70%	26	24.19 × 10^−4^	99.6%–100%

**Table 3:** Nucleotide mutations in VP1 gene from different passages of HY12 viruses.

Change		P2		P20		P40		P60		P80		P100	
Nucleotide	Amino Acid	Clone No	Proportion	Clone No	Proportion	Clone No	Proportion	Clone No	Proportion	Clone No	Proportion	Clone No	Proportion
72A > G	A24A	2	10%	0	/	0	/	0	/	0	/	0	/
272G > A	R91H	0	/	0	/	19	95%	19	95%	20	100%	20	100%
456G > A	P152P	0	/	0	/	16	80%	20	100%	20	100%	20	100%
609T > C	T203T	0	/	0	/	0	/	0	/	0	/	3	15%
652C > T	L218L	0	/	0	/	0	/	0	/	0	/	5	25%
723T > C	H240H	0	/	0	/	0	/	0	/	12	60%	0	/

**Table 4:** Nucleotide mutations in VP2 gene from different passages of HY12 viruses.

Change		P2		P20		P40		P60		P80		P100	
Nucleotide	Amino Acid	Clone No	Proportion	Clone No	Proportion	Clone No	Proportion	Clone No	Proportion	Clone No	Proportion	Clone No	Proportion
442C > G	Q148E	0	/	0	/	0	/	0	/	0	/	15	75%
667T > C	L223L	0	/	0	/	0	/	0	/	0	/	2	10%

## Data Availability

No new data were created or analyzed in this study. Data sharing is not applicable to this article.
